# Comparison of the Luminex® NxTAG® Gastrointestinal Pathogen Panel to traditional diagnostic methods for detecting diarrhoea-associated gastroenteritis

**DOI:** 10.1099/jmm.0.002089

**Published:** 2025-11-04

**Authors:** Kym Wilson, Paul Beckett, Michael Collins

**Affiliations:** 1 Microbiology Department, Derbyshire Pathology, Chesterfield Royal Hospital, Calow Chesterfield, Derbyshire, UK

**Keywords:** coinfection, diagnostics, gastroenteritis, multiplex reverse transcriptase PCR (RT-PCR), syndromic testing

## Abstract

**Introduction.** Gastrointestinal infections remain a leading cause of morbidity and mortality within the UK.

**Hypothesis.** The Luminex^®^ NxTAG^®^ Gastrointestinal Pathogen Panel (NxTAG GPP) multiplex reverse transcriptase PCR assay performs equivalently to standard-of-care diagnostic approaches.

**Aim.** To compare the analytical performance of the NxTAG GPP assay versus routine diagnostic testing methods in a district general hospital setting.

**Methodology.** Gastrointestinal pathogens in 159 faecal specimens from hospital inpatients and outpatient clinics were comparatively analysed using the NxTAG GPP assay versus traditional culture, enzyme immunoassay and molecular methods.

**Results.** Positive results were detected in 45 out of 159 specimens (28.3%) by NxTAG GPP, which was a higher positivity rate when compared with traditional diagnostic methods which detected 31 out of 159 (19.5%) positive infections (*P*=0.087 by Fisher’s exact test). Infections were caused by a single organism in 40 out of 45 (88.9%) cases, but 5 out of 45 (11.1%) infections detected were due to coinfections. No coinfections were detected by traditional methods. *Campylobacter* Group was the most common enteropathogen detected with 15 out of 52 (28.9%) infections. Viruses caused 26.9% of infections, including 15.4% being norovirus. Overall sensitivity, specificity and accuracy for the NxTAG GPP assay were 97.6%, 99.7% and 99.5%, respectively, for enteropathogenic bacteria and viruses detected during this study. No parasites were detected during this study and were not included in comparisons.

**Conclusions.** The NxTAG GPP assay demonstrated high sensitivity and specificity for identifying gastrointestinal pathogens, with comparable accuracy as more resource-intensive and time-consuming standard diagnostic approaches. The NxTAG GPP has the potential to enhance patient diagnosis, reduce turnaround time and improve clinical outcomes compared to routine diagnostic methods.

## Introduction

Gastroenteritis is caused by bacterial- and/or viral-mediated inflammation of the intestine and stomach linings, presenting with sudden-onset diarrhoea, sometimes with the presence of vomiting, fever and abdominal pain [1]. Symptoms begin hours to days after ingestion of pathogenic micro-organisms from contaminated food and water sources. Commonly encountered organisms associated with gastrointestinal infections include *Campylobacter*, *Salmonella*, norovirus and *Cryptosporidium*. In the UK, gastroenteritis is often mild and self-limiting and without long-term health effects. However, individuals who are considered to be vulnerable or high-risk (infants/elderly or immunocompromised) may have significant morbidity and mortality [2,3].

A study by the UK Food Standards Agency estimated 2.4 million gastrointestinal infections nationally in 2018, with ~16,400 of these requiring hospitalization and 222,000 involving presentation to general practices [4]. The large number of gastrointestinal infections incurs significant costs to the health system [2]. A contributing factor is the 48‒72 h required for patient diagnosis by traditional methods. Traditional diagnostic stool analysis of outpatients at Chesterfield Royal Hospital (the study site) is to culture for pathogen identification using selective agar, which can take 24‒48 h, depending on the organism. Antibiotic susceptibility analysis may take a further 24 h. Thus, the time constraints of culture-based pathogen identification carry a risk that optimal antimicrobial therapy selection is delayed. Also, suboptimal interim antimicrobial agents may cause antibiotic-associated infections including *Clostridium difficile* [5,7], which accounts for 10–25% of antibiotic-associated diarrhoea, or haemolytic uraemic syndrome with *Escherichia coli* O157 infection [8,10]. Rapid identification of the causative pathogen allows immediate selection and initiation of the most appropriate medical therapy, thereby optimizing patient outcomes.

Rapid and accurate identification of gastroenteritis-causative organisms is crucial to guide the most effective treatment to shorten the disease course and ensure that isolation methods are initiated if appropriate. Improved turnaround time also helps in identifying and tracking outbreak/epidemic situations, ensuring fast infection control measures and effective antimicrobial therapy if required. One advantage of traditional culture methods is that a viable organism is grown, which allows further testing such as antimicrobial susceptibility testing or speciation/typing [11]. However, the time to results is often >48 h, and routine testing methods require more stool (1‒2 g) than molecular methods. Also, traditional culture methods only identify a select number of organisms (Table 1). Culture methods require experienced staff with the ability to differentiate between normal faecal flora and potential pathogens. Additional enzyme immunoassays (EIAs), which are fast and easy to use, may be included in routine testing but only detect antigens for specific organisms, such as *C. difficile* or rotavirus, and can become costly [11]. Additionally, some pathogens are difficult or impossible to grow in laboratory conditions [12]. These examples highlight the importance of developing sensitive, comprehensive multiplex molecular technologies that quickly and simultaneously evaluate the presence of multiple gastrointestinal pathogens in limited-volume stool samples [12].

**Table 1.** Detectable organisms between methods

Organism	NxTAG GPP	Routine/culture*	BD MAX Enteric Bacterial Panel
*Campylobacter* Group	**√**	**√**	**√**
*C. difficile* (toxin A/B)	**√**	**•**	
ETEC (LT/ST)	**√**	**√**	**√**
STEC (*stx1*/*stx2*)	**√**	**√**	**√**
*Shigella* spp./EIEC	**√**	**√**	**√**
*Salmonella* spp.	**√**	**√**	**√**
*Vibrio cholerae*	**√**	**•**	
*Yersinia enterocolitica*	**√**	**•**	
Adenovirus F40/41	**√**	**•**	
Astrovirus	**√**	**•**	
Norovirus GI/GII	**√**	**•**	
Rotavirus A	**√**	**•**	
Sapovirus GI/GII/GIV/GV	**√**		
*Cryptosporidium* Group	**√**	**√**	
*Entamoeba histolytica*	**√**	**•**	
*Giardia lamblia*	**√**	**√**	

*Organisms indicated by **•** may be isolated by culture/detected by routine tests but are only tested for if indicated by clinical details and macroscopic appearance of stool.

In our laboratory, outpatients are tested by standard culture, microscopy and/or EIA methods, whereas samples from inpatients are tested using a multiplex molecular platform, the BD Max^™^ Enteric Bacterial Panel (Becton, Dickinson and Company, Franklin Lakes, NJ) that detects five common potential pathogens (Table 1). This system has a 2 h turnaround time, and with the addition of other tests including norovirus and *C. difficile* EIAs, inpatient results can be available within 3 h. The shorter turnaround time aids patient treatment, shortens hospital stay and improves patient flow but is limited in the scope of gastrointestinal pathogens evaluated.

The NxTAG^®^ Gastrointestinal Pathogen Panel (NxTAG GPP) from Luminex (Austin, TX) is a qualitative bead-based multiplex test for the molecular detection and identification of 16 gastrointestinal viral, bacterial and parasitic pathogens directly from human stool samples (Table 1). NxTAG GPP uses reverse transcriptase PCR (RT-PCR) combined with the Luminex tag sorting system on the MAGPIX instrument. This study is the first to evaluate the NxTAG GPP system for routine diagnostic use within a UK district general hospital laboratory and compare its clinical performance versus routine diagnostic methods.

## Methods

### Samples

Faecal samples from 159 patients presenting with gastrointestinal symptoms were consecutively analysed from samples received within the microbiology laboratory between 9 July and 7 September 2021 from both inpatients and outpatients at Chesterfield Royal Hospital, and GP surgeries from the surrounding community. Stools for *Helicobacter pylori*-only testing were excluded from the study, as were inpatient samples for *C. difficile* and/or norovirus testing only. Samples were received in sterile 100 ml universal containers without transport media; the macroscopic appearance was noted according to the Bristol Stool Chart. Specimens were immediately processed for standard-of-care analysis. Remaining specimens were aliquoted and stored at −70 °C to allow batch processing for NxTAG GPP analysis (16–45 samples per batch; maximum 10 days’ storage), and for follow-up culture studies. Study performance was exempt from institutional review board oversight due to the use of deidentified leftover samples from patients who had provided informed consent for sample testing and conformed to the tenets of the Declaration of Helsinki.

### Routine outpatient sample testing

Outpatient samples were sent for routine laboratory testing according to standard operating procedures in place. Briefly, samples were cultured on selective agar (Thermo Fisher Scientific, Waltham, MA) as follows: Xylose Lysine Deoxycholate agar for *Salmonella* spp. and *Shigella* spp. and Cefixime Tellurite Sorbitol MacConkey agar to isolate *E. coli* O157. Selenite broth was inoculated and incubated aerobically at 37 °C for up to 24 h and then subcultured onto chromogenic *Salmonella* agar and incubated for 24 more hours. *Campylobacter*-selective agar was used to isolate *Campylobacter* spp. in microaerophilic conditions at 42 °C for 48 h. Smears underwent Auramine staining and evaluation for *Cryptosporidium* spp. using fluorescence microscopy, with positives confirmed by Ziehl–Neelsen staining. Further tests were performed as indicated or requested, depending on clinical details and stool appearance: *C. difficile* EIA (C. Diff Quik Chek Complete^®^ kit, TechLab^®^, Blacksburg, VA), norovirus testing (RIDA^®^QUICK Norovirus EIA, R-Biopharm^®^, Darmstadt, Germany), rotavirus testing (Immunocard STAT!^®^ Rotavirus test, Meridian Bioscience, Cincinnati, OH) and ova and parasite examination on concentrated stool. Additionally, selective media such as Thiosulfate-Citrate-Bile salts-Sucrose agar and alkaline peptone water were set up on any samples suspected for *V. cholerae* or *V. parahaemolyticus*. Cefsulodin-Irgasan-Novobiocin agar was used for suspected *Yersinia* spp. Deoxycholate citrate agar was used to isolate any *Shigella* spp. These routine culture methods can take up to 48‒72 h for a complete result to be reported and authorized.

### Routine inpatient sample testing

Inpatient samples are routinely tested using the BD Max^™^ Enteric Bacterial Panel. This multiplex molecular panel detects five bacterial targets (Table 1). Approximately 10 µl of stool was inoculated into Sample Buffer and loaded onto the BD Max system per the manufacturer’s instructions [13]. Results were obtained after 2.5 h. Including additional testing for *C. difficile* and norovirus by EIA, the average turnaround time for inpatient results is ≈3 h.

### NxTAG GPP test method

For the NxTAG GPP assay, 100‒150 mg of formed stool or 100 µl of liquid stool was added to Bertin^®^ SK38 Soil Kit bead tubes (Bertin Corp., Bethesda, MD) containing 1 ml of NucliSENS^®^ easyMAG^®^ lysis buffer (bioMérieux^®^, Marcy-l'Étoile, France) with 20 µl of bacteriophage MS2 (internal control). A negative extraction control was also prepared for each run to ensure that no cross-contamination had occurred. Tubes were vortexed for 5 min, centrifuged at 20,800 RCF for 2 min, and 100 µl aliquots of each sample supernatant were used for nucleic extraction using the KingFisher^™^ Flex Purification System (Thermo Fisher Scientific, Bedford, OH) with the MVP-2wash-200-flex protocol, producing 200 µl of extracted nucleic acids. Thirty-five microlitres of each extracted sample were pipetted into the NxTAG GPP plate vessels containing lyophilized bead reagents, mixed, and the plate sealed for combined RT-PCR amplification and bead hybridization in an Eppendorf^®^ Mastercycler^®^ thermocycler (Eppendorf, Hamburg, Germany). Completed reaction plates were transferred to the MAGPIX^®^ instrument (Luminex), where the hybridized, tagged beads were sorted and read using SYNCT^™^ software. Signals generated by the tagged beads were analysed to create a qualitative summation for the 16 assay targets.

### Statistical analysis

Data are presented as means and numbers (per cent) of groups. Categorical variables were evaluated using Fisher’s exact test, with 2-tailed *P*-values<0.05 considered statistically significant, using GraphPad Prism v.9 (GraphPad Software, Boston, MA).

## Results

A total of 159 faecal samples were included in this study. Patient and sample characteristics are shown in Table 2. Male subjects accounted for 70 out of 159 (44.0%) of samples with 89 out of 159 (56.0%) received from females. The mean patient age was 57.2 years (range 2 months to 92 years). Most samples originated from outpatient clinics and GP surgeries (103 out of 159; 64.8%), with 56 out of 159 (35.2%) from inpatients. Most specimens were unformed (51 out of 159; 30.8%).

**Table 2.** Summary of patient and sample characteristics

Characteristic	No.	% of total
**Patient sex**		
Male	70	44.0
Female	89	56.0
**Age (years)**		
0–5	15	9.4
6–20	7	4.4
21–50	25	15.7
51–65	35	22.0
>65	77	48.4
**Patient status**		
Inpatient	56	35.2
Outpatient	103	64.8
Stool appearance*		
Formed	17	10.7
Semi-formed	45	28.3
Unformed	51	32.1
Liquid	45	28.3

*One of 159 not determined.

NxTAG GPP detected positive results in 28.3% (45 out of 159) of specimens, which was higher than the 18.9% (30 out of 159) of pathogen-positive specimens obtained by standard diagnostic testing (Table 3). While the NxTAG GPP assay-detected fraction was numerically greater than with standard diagnostic evaluation, the two methods were statistically comparable (*P*=0.064). With NxTAG GPP, a single pathogen was detected in 40 samples with coinfections detected in 5 cases (11% of positive specimens). A single coinfection was identified by standard laboratory diagnostic techniques (*P*=0.204). Of the 5 coinfections detected with NxTAG GPP, 2 pathogens were detected in 3 samples, and 3 pathogens were detected in 2 samples, for a total of 52 organisms detected by NxTAG GPP. A total of 31 infections were detected by combined standard-of-care methods (culture for outpatients and BD MAX Enteric Panel for inpatients) (Table 3).

**Table 3.** Pathogens detected by NxTAG GPP and standard-of-care* testing

	NxTAG GPP	SoC† (Culture and BD MAX)
Pathogen	Number (% of detections)	Number (% of detections)
**Bacteria**		
*Campylobacter* Group	15 (28.8%)	13 (41.9%)
*C. difficile* (toxin A/B)	14 (26.9%)	8 (25.8%)‡
ETEC (LT/ST)	2 (3.8%)	0
STEC (*stx1*/*stx2*)	4 (7.7%)	2 (6.5%)
*Salmonella* spp.	2 (3.8%)	0
*Y. enterocolitica*	1 (1.9%)	0
**Viruses**		
Adenovirus F40/41	1 (1.9%)	0
Astrovirus	2 (3.8%)	0
Norovirus GI/GII	8 (15.4%)	8 (25.8%)
Rotavirus A	2 (3.8%)	0
Sapovirus GI/GII/GIV/GV	1 (1.9%)	0
**Overall**	**52 (100%)**	**31 (100%)**

*Pathogens detected prior to discordant call resolution.

†Standard-of-care diagnosis includes culture-based methods and EIA for outpatients, and the BD Max Enteric Bacterial Panel and EIA for inpatients. Of the 31 SoC detections, 6 *Camplylobacter* and 1 STEC were detected in inpatients using the BD Max Panel.

‡Five patients with NxTAG GPP-positive *C. difficile* were not tested for *C. difficile* by SoC methods because stool was formed or semi-formed, according to institutional policies designed to minimize unnecessary testing.

Of NxTAG GPP-detected bacterial pathogens, *Campylobacter* Group comprised 28.8% (15 out of 52), *C. difficile* comprised 26.9% (14 out of 52), Shiga toxin-producing *E. coli* (STEC) and enterotoxigenic *E. coli* (ETEC) made up 7.7% (4 out of 52) and 3.8% (2 out of 52), respectively, *Salmonella* spp. made up 3.8% (2 out of 52) and 1 case of *Y. enterocolitica* (1.9%) was detected. Viruses accounted for 26.9% (14 out of 52) of the total enteropathogens detected by NxTAG GPP, with norovirus (15.4%; 8 out of 52) being the most abundant; by standard-of-care testing, viruses accounted for 25.8% (8 out of 31) of pathogen detections, all of which were norovirus. Astrovirus and rotavirus each accounted for 3.9% (2 out of 52) of infections, and adenovirus F40/41 and sapovirus were found in 1 case each by NxTAG GPP but not by standard diagnostics. The initial invalid rate for the NxTAG GPP assay was 7 out of 159 (4.4%), presumably due to PCR inhibition, because after 1 : 10 dilution and retesting, a valid result was obtained for all 7 specimens.

No *Shigella* spp./enteroinvasive *E. coli* (EIEC), *V. cholerae*, *Cryptosporidium* Group, *E. histolytica* or *G. lamblia* were detected and were not included in sensitivity and accuracy comparisons. Adenovirus F40/41, astrovirus and sapovirus detections not confirmed by in-house methods or by a reference laboratory were also excluded from performance comparisons.

Performance summaries for NxTAG GPP vs standard-of-care testing are shown in Table 4. NxTAG GPP demonstrated 100% sensitivity for *Campylobacter* Group, *C. difficile*, ETEC, STEC, *Y. enterocolitica* and rotavirus. True positive detections accounted for 41, and false positive detections accounted for 8 (2 *Campylobacter* Group, 1 STEC, 2 *Salmonella* spp., 1 astrovirus, 1 norovirus and 1 sapovirus) of 2,544 total results. Specificity was 98.6% for *Campylobacter* Group, 98.7% for *Salmonella* spp., 99.4% for STEC, 99.3% for norovirus, 99.4% for astrovirus and sapovirus and 100% for the remaining pathogens. The accuracy of NxTAG GPP for the detected pathogens was 100% for *C. difficile*, ETEC, *Y. enterocolitica* and rotavirus. NxTAG GPP demonstrated an overall sensitivity of 97.6%, an overall specificity of 99.7% and an overall accuracy of 99.5%.

**Table 4.** Performance characteristics of each test method*

NxTAG GPP				Routine diagnostic tests		
Pathogen	Sensitivity (%)	Specificity (%)	Accuracy (%)	Sensitivity (%)	Specificity (%)	Accuracy (%)
**Bacteria**						
*Campylobacter* Group	100	98.6	98.7	100	100	100
*C. difficile* (toxin A/B)	100	100	100	88.9	100	99.0
ETEC (LT/ST)	100	100	100	50	100	99.4
STEC (*stx1*/*stx2*)	100	99.4	99.4	100	100	100
*Shigella* spp./EIEC	nd†	100	nd	nd	100	nd
*Salmonella* spp.	nd	98.7	98.7	nd	100	nd
*V. cholerae*	nd	100	nd	nd	100	nd
*Y. enterocolitica*	100	100	100	0	100	nd
**Viruses**						
Adenovirus	nd	100	nd	‡		
Astrovirus	nd	99.4	nd			
Norovirus GI/GII	87.5	99.3	98.7	100	99	99.1
Rotavirus A	100	100	100	0	100	98.1
Sapovirus	nd	99.4	nd			
*Cryptosporidium* Group	nd	100	nd	nd	100	nd
*E. histolytica*	nd	100	nd	nd	100	nd
*G. lamblia*	nd	100	nd	nd	100	nd
**Overall**	97.6	99.7	99.5	85.2	99.9	99.0

*Includes results that could be confirmed by another method only.

†nd, not determined.

‡Grey shading indicates not tested.

The standard diagnostic methods demonstrated an overall sensitivity of 85.2% (culture and BD MAX results combined), which was lower than the sensitivity with NxTAG GPP (Table 4). This difference was largely driven by a single false positive for ETEC by routine culture. Overall specificity with routine methods was comparable to NxTAG GPP at 99.9% (and 100% sensitivity for inpatients tested with the BD MAX assay; not shown). Overall accuracy was also comparable to NxTAG GPP at 99.0% for the traditional tests (99.6% for BD MAX alone).

## Discussion

The increase in the availability of multiplex PCR technology has greatly improved gastrointestinal pathogen identification and detection. This study demonstrated excellent clinical performance of the NxTAG GPP assay, showing 97.6% sensitivity, 99.7% specificity and 99.5% accuracy for identifying 16 common gastrointestinal pathogens. The NxTAG GPP assay had a numerically higher positivity rate (28.3%) compared with routine testing methods (18.9%), although this difference was not significant.

While this report was under construction in 2025, two publications emerged on NxTAG GPP performance [14,15]. An initial evaluation of 505 stool specimens from patients with gastroenteritis in a German medical centre revealed the NxTAG GPP assay to have comparable or even superior performance to standard diagnostic tests (i.e. selective culture/enrichment with microscopy and/or PCR follow-up) [14]. Outstanding NxTAG GPP assay performance and time/resource savings were the drivers for that medical centre to have adopted the multiplex test as their routine stool analysis method versus traditional microbiological diagnostic approaches [14]. Excellent overall NxTAG GPP assay performance was confirmed in subsequent analysis of 2,462 stool samples by the same group, although potential false positives for *Salmonella* spp. with NxTAG GPP prompted the addition of a confirmatory PCR assay when this pathogen was detected. The authors speculated that this may be due to suboptimal *Salmonella* primer design. Nonetheless, this modification only modestly impacted diagnostic efficiency because the rate of *Salmonella*-positive samples encountered and requiring corroboration was low (3.1% of tested specimens) [14]. We also encountered two false positives for *Salmonella* spp. Another research group at a Spanish medical centre compared the performance of the NxTAG GPP assay to another multiplex molecular diagnostic assay, the Allplex^®^ assay (Seegene, Seoul, South Korea), that evaluates 25 potential pathogens [15]. Using 196 stool samples from patients with gastroenteritis, both assays demonstrated high overall concordance, with Negative Percent Agreement consistently above 95% and overall kappa values>0.8 for most pathogens. Average Positive Percent Agreement was >89% for nearly all targets [15]. Discrepancies were primarily observed for *Salmonella* spp. (κ=0.64) and *Cryptosporidium* spp. (κ=0.73). Factors responsible for these differences may include variations in the specific genomic regions targeted and differences in primer design between the two assays [15]. Additionally, some genomic regions can exhibit polymorphisms, which are particularly common in certain micro-organisms like *Cryptosporidium* spp. [16]. Whereas the NxTAG GPP assay measures all 16 targets in a single reaction tube, the Allplex assay requires 4 tubes per sample to cover all target pathogens, with an associated increase in operator hands-on time requirements.

Multiplex molecular diagnostic assays such as the NxTAG GPP assay offer significant time savings over traditional culture methods that can require several days to provide initial results. The NxTAG GPP assay represents an improvement over its predecessor, the xTAG GPP assay, insofar as the pathogen target panel has been expanded and the workflow has been streamlined by consolidating the multiplex RT-PCR and bead hybridization tasks into a single step [17,18]. The elimination of a sample-handling step reduces the likelihood of introducing environmental contaminants. Regarding targeted pathogens, *E. coli* O157 is no longer included in the updated NxTAG GPP panel but will be captured as an STEC target. While *E. coli* O157 carries an important risk of antibiotic-associated haemolytic uraemic syndrome, other non-O157 STEC serogroups can cause severe disease, including HUS [19]. Primer sets to identify astrovirus and sapovirus have been newly added.

Performance of the NxTAG GPP assay in the current study was comparable to or improved versus prior evaluations using the antecedent xTAG GPP assay. For example, in a multinational evaluation of 901 stool samples from patients with gastroenteritis symptoms, predecessor xTAG GPP assay sensitivity determined for 12 of 15 pathogen targets was 94.3% overall, with specificity across all 15 targets at 98.5% [20]. In a US study of 211 subjects using the earlier xTAG GPP assay, an overall sensitivity determined for 11 of 15 targets was 96.4% [21]. Sensitivity in the current study with the contemporary NxTAG GPP assay was 97.6%.

Assay refinement has cut the typical specimen-to-results time from an estimated 5 h with the older xTAG GPP assay to ~4 h with NxTAG GPP, well below the multiple days required for culture-based answers. Compared with standard culture-centric diagnostics, the xTAG/NxTAG GPP assays can reduce technician hands-on time by 75% and total assay time by over 90% [21]. A UK health economics study calculated that, compared with conventional testing, multiplex molecular testing with xTAG GPP increases the positive infection detection rate two-fold, cuts actionable diagnostic time almost in half and reduces isolation days and associated costs by 34% [22]. Thus, beyond the initial expenses required for equipment and laboratory staff training, adoption of the high-performance multiplex NxTAG GPP molecular diagnostic assay may provide appreciable long-term cost savings versus culture-based assessment by reducing technician workload while permitting rapid and simultaneous generation of comprehensive gastrointestinal pathogen profiles in up to 96 patient specimens per assay plate.

Multiplex PCR-based assays like NxTAG GPP can simultaneously detect multiple infective micro-organisms in stool samples, thereby identifying pathogens that might be missed by targeted single-pathogen assays and remain untreated. Coinfections occurred in 11.1% of positive specimens, including two specimens in which three pathogens were identified; this multiple pathogen rate is similar to the 14.3% coinfection rate recently reported by others with NxTAG GPP and the 12.8% coinfection rate with another multiplex PCR panel [15]. In the current study, routine culture identified a single coinfection incident. One limitation of multiplex PCR testing is that it detects nucleic acids only and cannot differentiate viable versus nonviable organisms; thus, organisms may be detected by PCR even if they are not the current cause of infection symptoms. For example, one patient was identified by NxTAG GPP to have a coinfection with *C. difficile* and norovirus; however, the patient was under 2 years old, so it was interpreted that the symptoms were probably due to norovirus, and they were an asymptomatic carrier of *C. difficile*. Supporting this idea, up to 84% of young children can be asymptomatic colonizers of this organism [23]. In older children and adults, accurate identification of *C. difficile* infection, whether alone or in a coinfection scenario, is essential because this organism is the most common cause of nosocomial antibiotic-associated diarrhoea [24] and, with a case-fatality rate of 12.9%, was responsible for an estimated 2,164 deaths in the UK during fiscal year 2023‒2024 [25].

The NxTAG GPP had an initial invalid rate of 4.4%, which occurs when the internal control is not detected, mainly due to the presence of amplification inhibitors that lead to false-negative results. Invalid samples were retested following 1 : 10 dilution of extracted nucleic acid per manufacturer instructions and provided a valid result in all cases. Rerunning invalid samples is a cost pressure, but the small number of invalids was still a cost-benefit to the patient since the results of the reruns could still be retrieved in less time than traditional methods. The cases where *Y. enterocolitica*, STEC and ETEC were detected by the NxTAG GPP assay were considered very significant because these infections were not detected during routine diagnostic testing. *Y. enterocolitica* testing is typically only undertaken if indicated in the clinical details or specifically requested by a clinician; however, the other two pathogens were missed during routine culture methods using selective agar.

While eight of the specimens positive for *C. difficile* by NxTAG GPP were also positive by EIA, five were not assessed in routine testing because the macroscopic appearance of the sample (formed or semi-formed) did not meet laboratory policy requirements for *C. difficile* testing. *C. difficile* infection causes loose, watery stool, so only testing of liquid and unformed samples is considered necessary [26]. The NxTAG GPP returned two false-positive results for *Salmonella* spp., one of which was identified as *Citrobacter freundii* by MALDI-TOF. This may indicate cross-reactivity or sequence homology in the region detected by NxTAG GPP, but unfortunately, insufficient remaining sample precluded further testing to resolve this discrepancy. While the NxTAG GPP assay provides greatly improved performance over conventional culture-based diagnostic testing [14,15], additional design optimization for the detection of *Salmonella* spp. may reduce or eliminate potential false positives for this organism.

Study limitations include the analysis of stool samples collected from a single site in the UK, which restricts the generalizability of study findings to a similar population. While this was a feasibility study to investigate assay utility potential at our medical centre, the number of specimens evaluated (*N*=159) meant that several assay targets were not observed, which limited more rigorous statistical analysis. While the proportions of both total pathogens and coinfections identified by NxTAG GPP were greater than by culture-based analysis, with differences approaching statistical significance, a larger sample size would enhance these comparisons. Because specimens were collected during the northern-hemisphere summertime months of July‒September and many enteropathogenic organisms display seasonal variation [27,28], this may explain the relatively high incidence of *Campylobacter* spp. and low incidences of enteroviral infections identified. A potential disadvantage of using PCR technology for diagnosing gastroenteritis is that it does not necessarily identify a viable organism, so the organism must be retrieved if further testing is needed, such as antimicrobial susceptibility or speciation/typing [11].

## Conclusion

This study demonstrated the NxTAG GPP assay’s high sensitivity, specificity and accuracy for simultaneously interrogating stool samples for multiple diarrhoeal pathogens. Compared to traditional culture-based techniques, the NxTAG GPP assay reduces diagnostic turnaround time, therefore providing faster clinical input for swift patient treatment. Together with the high-throughput assay format, we deem this assay suitable for use in the clinical diagnostic laboratory. Future studies with large, diverse sample sets and conducted over a longer period of time are needed to fully assess the capabilities of the NxTAG GPP assay and to permit more rigorous statistical comparisons of performance versus culture-based diagnostics.

## Abbreviations

EIAs: enzyme immunoassays
EIEC: enteroinvasive *Escherichia coli*
ETEC: enterotoxigenic *Escherichia coli*
NxTAG GPP: NxTAG^®^ Gastrointestinal Pathogen Panel
STEC: Shiga toxin-producing *Escherichia coli*
